# Post‐exercise intramuscular O_2_ supply is tightly coupled with a higher proximal‐to‐distal ATP synthesis rate in human tibialis anterior

**DOI:** 10.1113/JP280771

**Published:** 2021-01-13

**Authors:** Linda Heskamp, Franciska Lebbink, Mark J. van Uden, Marnix C. Maas, Jurgen A. H. R. Claassen, Martijn Froeling, Graham J. Kemp, Andreas Boss, Arend Heerschap

**Affiliations:** ^1^ Department of Medical Imaging/Radiology Radboud university medical center Nijmegen The Netherlands; ^2^ Department of Geriatrics Radboud university medical center Nijmegen The Netherlands; ^3^ Department of Radiology University Medical Center Utrecht Utrecht The Netherlands; ^4^ Department of Musculoskeletal and Ageing Science University of Liverpool Liverpool UK

**Keywords:** ^31^P magnetic resonance spectroscopy, magnetic resonance imaging, oxidative metabolism, phosphocreatine recovery, skeletal muscle

## Abstract

**Key points:**

- The post‐exercise recovery of phosphocreatine, a measure of the oxidative capacity of muscles, as assessed by ^31^P MR spectroscopy, shows a striking increase from distal to proximal along the human tibialis anterior muscle.
- To investigate why this muscle exhibits a greater oxidative capacity proximally, we tested whether the spatial variation in phosphocreatine recovery rate is related to oxygen supply, muscle fibre type or type of exercise.
- We revealed that oxygen supply also increases from distal to proximal along the tibialis anterior, and that it strongly correlated with phosphocreatine recovery. Carnosine level, a surrogate measure for muscle fibre type was not different between proximal and distal, and type of exercise did not affect the gradient in phosphocreatine recovery rate.
- Taken together, the findings of this study suggest that the post‐exercise spatial gradients in oxygen supply and phosphocreatine recovery are driven by a higher intrinsic mitochondrial oxidative capacity proximally.

**Abstract:**

Phosphorus magnetic resonance spectroscopy (^31^P MRS) of human tibialis anterior (TA) revealed a strong proximo‐distal gradient in the post‐exercise phosphocreatine (PCr) recovery rate constant (*k*_PCr_), a measure of muscle oxidative capacity. The aim of this study was to investigate whether this *k*_PCr_ gradient is related to O_2_ supply, resting phosphorylation potential, muscle fibre type, or type of exercise. Fifteen male volunteers performed continuous isometric ankle dorsiflexion at 30% maximum force until exhaustion. At multiple locations along the TA, we measured the oxidative PCr resynthesis rate (*V*_PCr_ = *k*_PCr_ × PCr depletion) by ^31^P MRS, the oxyhaemoglobin recovery rate constant (*k*_O2Hb_) by near infrared spectroscopy, and muscle perfusion with MR intravoxel incoherent motion imaging. The *k*_O2Hb_, *k*_PCr_, *V*_PCr_ and muscle perfusion depended on measurement location (*P* < 0.001, *P* < 0.001, *P* = 0.032 and *P* = 0.003, respectively), all being greater proximally. The *k*_O2Hb_ and muscle perfusion correlated with *k*_PCr_ (*r* = 0.956 and *r* = 0.852, respectively) and *V*_PCr_ (*r* = 0.932 and *r* = 0.985, respectively), the latter reflecting metabolic O_2_ consumption. Resting phosphorylation potential (PCr/inorganic phosphate) was also higher proximally (*P* < 0.001). The surrogate for fibre type, carnosine content measured by ^1^H MRS, did not differ between distal and proximal TA (*P* = 0.884). Performing intermittent exercise to avoid exercise ischaemia, still led to larger *k*_PCr_ proximally than distally (*P* = 0.013). In conclusion, the spatial *k*_PCr_ gradient is strongly associated with the spatial variation in O_2_ supply. It cannot be explained by exercise‐induced ischaemia nor by fibre type. Our findings suggest it is driven by a higher proximal intrinsic mitochondrial oxidative capacity, apparently to support contractile performance of the TA.

## Key points

- The post‐exercise recovery of phosphocreatine, a measure of the oxidative capacity of muscles, as assessed by ^31^P MR spectroscopy, shows a striking increase from distal to proximal along the human tibialis anterior muscle.
- To investigate why this muscle exhibits a greater oxidative capacity proximally, we tested whether the spatial variation in phosphocreatine recovery rate is related to oxygen supply, muscle fibre type or type of exercise.
- We revealed that oxygen supply also increases from distal to proximal along the tibialis anterior, and that it strongly correlated with phosphocreatine recovery. Carnosine level, a surrogate measure for muscle fibre type was not different between proximal and distal, and type of exercise did not affect the gradient in phosphocreatine recovery rate.
- Taken together, the findings of this study suggest that the post‐exercise spatial gradients in oxygen supply and phosphocreatine recovery are driven by a higher intrinsic mitochondrial oxidative capacity proximally.

## Introduction

Muscle contraction requires energy in the form of ATP, whose production is dominated by oxidative phosphorylation in the mitochondria. In any temporary mismatch between ATP supply and use, such as at the onset of exercise, the phosphocreatine (PCr) energy buffer system comes into play, re‐phosphorylating ADP to ATP at the expense of PCr, catalysed by creatine kinase. When the ATP demand of force‐generation ceases, the PCr pool is replenished by oxidative phosphorylation. The post‐exercise recovery rate constant of PCr (*k*_PCr_), which can be measured using dynamic phosphorus MR spectroscopy (^31^P MRS), can be interpreted as a correlate and marker of the muscle's oxidative capacity, providing that minimal pH changes occur in the preceding exercise period (Taylor *et al*. 1983; Meyer, 1988; McCully *et al*. 1993; Kemp *et al*. 2015). In numerous studies *k*_PCr_ varies as expected between trained *vs*. untrained subjects, young *vs*. elderly, and healthy subjects *vs*. subjects with mitochondrial myopathies or type II diabetes (Arnold *et al*. 1985; Phielix *et al*. 2008; Larsen *et al*. 2009; Fleischman *et al*. 2010). Furthermore, *k*_PCr_ is higher in muscles containing predominantly oxidative type I fibres than in muscles in which glycolytic type II fibres are dominant (Söderlund and Hultman, 1991; Kushmerick *et al*. 1992; Yoshida *et al*. 2013). In all these situations, measures derived from post‐exercise PCr recovery kinetics correlate with *ex vivo* measures of mitochondrial function or content (Taylor *et al*. 1983; Meyer, 1988; McCully *et al*. 1993; Kemp *et al*. 2015). In ^31^P MRS and biopsy studies of mitochondrial function it is conventionally assumed that spatial/anatomical gradients within a single muscle body can be neglected. Strikingly, though, we reported that *k*_PCr_ does vary within a single muscle: following continuous isometric exercise, *k*_PCr_ was significantly greater in the proximal part of the tibialis anterior (TA) than in the distal part (Boss *et al*. 2018).

Despite the dominant effects of mitochondrial activity and content, PCr recovery may also reflect extra‐mitochondrial factors (Taylor *et al*. 1983; Meyer, 1988; McCully *et al*. 1993; Kemp *et al*. 2015). The most important of these is perhaps the vascular supply of O_2_: PCr recovery can be markedly slowed when O_2_ supply is impaired, e.g. in peripheral vascular disease (Harris *et al*. 1976; Kemp *et al*. 2015). Similar influences are seen in normal physiology: in healthy subjects muscle perfusion, measured by MR, correlates with *k*_PCr_ (Carlier *et al*. 2006). However, whether this coupling between *k*_PCr_ and O_2_ supply also holds when *k*_PCr_ varies spatially within a single muscle is unknown.

The first aim of this study was to assess whether processes involved in O_2_ supply vary spatially along the TA and to what extent this is related to the spatial gradient of post‐exercise PCr recovery. We addressed this by combining ^31^P MRS with two complementary techniques, near infrared spectroscopy (NIRS) and intravoxel incoherent motion imaging (IVIM). NIRS measures changes in oxyhaemoglobin (O_2_Hb), which reflects the temporary imbalance between O_2_ supply and O_2_ use, and IVIM measures parameters that reflect muscle perfusion, which is a main determinant of O_2_ supply.

Mitochondrial function has also been related to muscle fibre type and to resting‐muscle values of free energy of ATP hydrolysis (or phosphorylation potential) (Meyer, 1988; Söderlund and Hultman, 1991; Schiaffino and Reggiani, 2011; Kemp *et al*. 2015). Therefore, our second aim was to investigate whether muscle fibre type and phosporylation potential varied along the length of the TA with MRS. While the definitive assessment of fibre type proportions requires biopsy, a conveniently non‐invasive surrogate biomarker for fibre types in healthy muscles is carnosine content (Harris *et al*. 1998), which can be measured by ^1^H MRS (Baguet *et al*. 2011). The PCr/Pi ratio, a surrogate marker for phosphorylation potential, can readily be obtained from ^31^P MR spectra of resting muscle (Meyer *et al*. 1985; Kushmerick *et al*. 1992; Takahashi *et al*. 1996; Kemp *et al*. 2015).

Finally, to examine the possibility that the observed k_PCr_ gradient is due to spatial variations in the degree of ischaemia caused by continuous isometric exercise, we performed ^31^P MRS measurements following intermittent isometric exercise, in which muscle perfusion is maintained during the relaxation phase.

## Methods

### Ethical approval

This study was conducted according to the principles of the *Declaration of Helsinki* (version October 2013) and the Medical Research Involving Human Subjects Act (WMO), except for registration in a database. It was approved by the local medical ethical committee CMO Arnhem‐Nijmegen (NL60135.091.16 and NL58944.091.16), and prior written informed consent was obtained from all subjects.

### Subjects and study design

We recruited 20 healthy male subjects aged 18–35 years with BMI 18–25 kg/m^2^. Exclusion criteria were contra‐indications for MRI scanning or a history of muscular disease. Of the 20 participants, 15 subjects performed continuous isometric ankle dorsiflexion and five subjects performed intermittent isometric ankle dorsiflexion. Daily‐life activity was determined for the subjects performing the continuous exercise using a validated questionnaire (Craig *et al*. 2003).

**Figure 1:**
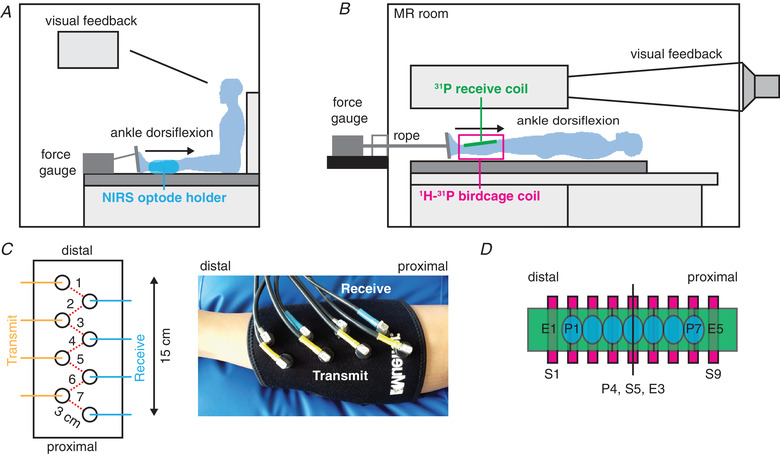
Schematic overview of the experimental approach *A*, set‐up for the NIRS measurement. The subject's foot was placed in a shoe attached to a pedal connected to a force gauge. The place of the NIRS optode holder is indicated in blue. *B*, set‐up for the MRI measurements. The subject's foot was placed in a shoe attached to a pedal and connected with a rope to a force gauge outside the scanner room. The subject received visual feedback by a projecter placed in the control room. *C*, overview of the placement of the four transmit and four receive optodes (all 3 cm apart) for the NIRS measurement, allowing oxyhaemoglobin assessment at seven positions along the muscle. *D*, schematic overview of the positioning of the seven NIRS measurement locations (blue, P1 to P7), the nine diffusion‐weighted slices (pink, S1 to S9) and the five ^31^P coil elements (green, E1 to E5). The middle positions (P4, S5, and E3) were centred at one third the distance between the fibula head and lateral malleolus, near the muscle belly. The 20 cm long ^31^P array coil covers the voluminous part of the tibialis anterior.

For the continuous exercise, the subjects underwent two experimental sessions; the first for NIRS and the second for MR measurements (IVIM and ^31^P MRS). For the intermittent exercise, we conducted only ^31^P MRS without IVIM and NIRS measurements. The subject's right foot was placed in a custom‐built MR‐compatible ergometer connected to a digital force gauge (Sauter FL 500, Balingen, Germany; Figs. 1*A*/1*B*) (Boss *et al*. 2018). At the start of each session, maximum voluntary contraction (MVC) for ankle dorsiflexion was determined as the best of three attempts. This was followed by 15 min rest to allow restoration of resting‐muscle perfusion and PCr levels. The continuous isometric ankle dorsiflexion exercise was performed at 30% MVC until exhaustion (approximately 2 to 5 min), and the intermittent isometric ankle dorsiflexion (frequency: 0.5 Hz) started at 10% MVC and incrementally increased with 10% MVC every 30 s until exhaustion (till approximately 60%–70% MVC).

### Data acquisition

#### NIRS

Concentration changes in O_2_Hb were measured simultaneously at seven positions along the TA using an 8‐channel continuous‐wave NIRS system (OxyMon MK III, Artinis Medical System, Elst, The Netherlands). We used three wavelengths (765, 857 and 859 nm) and a 50 Hz sampling frequency. The dependent differential path length factor (DPF), which accounts for the increased distance travelled by the light due to scattering, was set at 4.0 to express the O_2_Hb signal in micromol per litre (Ferrari *et al*. 1992). However, the use of this DPF is only for presentational convenience, because the scattering properties of the tissue are likely to vary along the TA and we have therefore only assessed the kinetics of the O_2_Hb signal, and not the amplitude changes. The total muscle coverage was 15 cm, with the seven positions centred 2.5 cm apart (Figs. 1*C*/1*D*). The transmitter–receiver inter‐optode distance was 3 cm, resulting in a measurement depth of ∼1.5 cm (McCully and Hamaoka, 2000). Data were acquired during 5 min rest, the exercise period, and 15 min recovery.

#### MR measurements

The MR measurements were performed on a 3T MR system (Magnetom PrismaFit, Siemens, Erlangen, Germany). In the case of the continuous exercise, the subject performed the exercise twice; first to assess muscle perfusion with IVIM and second to measure the PCr recovery with ^31^P MRS. The muscle perfusion measurement was performed first because the recovery of perfusion to resting post‐exercise values is considerably longer than recovery of ^31^P metabolite levels. There was at least 45 min between the two exercise bouts; this is sufficient time for perfusion and the phosphorous metabolites to recover to pre‐exercise values.

##### IVIM

MR images were acquired with a 15‐channel Tx‐Rx knee coil. We recorded nine transversal slices with a diffusion‐weighted spin‐echo sequence using echo‐planar imaging read‐out and spectral adiabatic inversion recovery fat suppression (repetition time (TR): 2000 ms, echo time (TE): 40 ms, field of view (FOV): 176 × 176 mm, voxel size 2.75 × 2.75 × 10 mm, number of slices: 9, slice gap 11.3 mm, receiver bandwidth 2440 Hz/pixel, acquisition time: 1 min 18 s). A total of 13 b‐values were obtained (0, 5, 10, 15, 20, 40, 60, 80, 100, 150, 200, 400 and 600 s mm^−2^) in three orthogonal directions. The slices covered a length of 18.4 cm along the proximo‐distal axis (Fig. 1*D*). We acquired four repetitions of the diffusion‐weighted scan before the start of the exercise, and 12 repetitions after the exercise.

##### ^31^P MRS

^31^P MR spectra were obtained with a custom‐built ^31^P phased array probe (van Uden *et al*. 2012; Boss *et al*. 2018) for signal reception, consisting of five individual coil elements (size: 4 × 4.5 cm (estimated measurement depth: ∼2 cm), total size: 4 × 20 cm, overlap of elements for decoupling, Fig. 1*D*). We combined this receive coil with a commercially available ^1^H/^31^P birdcage coil (Rapid, Rimpar, Germany) for homogeneous phosphorus excitation. This set‐up enabled us to receive free induction decays with a high signal‐to‐noise ratio (SNR) at five positions along the TA. The examination started with the acquisition of anatomical T1‐weighted images in resting muscle to verify correct placement of the ^31^P phased array probe (TR: 685 ms, TE: 12 ms, flip angle: 140°, FOV: 176 × 176 mm, voxel size: 0.92 × 0.92 × 10 mm, slice gap 11.3 mm, number of slices: 9, number of averages (NA): 2, turbo spin‐echo factor: 3). If the two fish oil capsules, which are attached at the centre of the two outermost elements of the ^31^P probe, did not appear directly above the TA, the ^31^P probe was repositioned. Next, we performed 2D ^31^P MR imaging in the transversal plane with a gradient echo sequence (centre frequency on PCr, TR: 1500 ms, TE: 10 ms, NA: 6, FOV: 176 × 176 mm, matrix size: 16 × 16). A 20 cm thick axial slice covering the TA perpendicular to the coil was selected so that for each coil element a 2D transversal ^31^P MR image is obtained of the intersection of its sensitive area and this slice. Thereafter, sequential ^31^P MR spectra were obtained (TR: 2.06 s, 48° Ernst angle excitation, ^1^H‐^31^P NOE enhanced). We collected for each of the five coil elements ^31^P MRS spectra at rest (six averages per spectrum) to determine PCr/Pi, which was followed by the exercise protocol. The exercise protocol consisted of 1 min rest, the continuous or intermittent isometric exercise until exhaustion, and the recovery period. Throughout this rest, exercise and recovery period ^31^P MR spectra were continuously recorded, with two averages per spectrum, for 20 min 36 s.

##### Carnosine

*S*ingle voxel ^1^H MR spectra were acquired during rest with the ^31^P/^1^H birdcage coil in six of the volunteers using an sLASER sequence for voxel localization (TR: 3000 ms, TE: 33 ms, NA = 144, bandwidth: 1200 Hz) with and without water suppression. Two voxels of 17 × 17 × 60 mm were measured, one in the distal and the other in the proximal part of the muscle, avoiding contamination by subcutaneous fat and the extensor digitorum.

### Data processing

Data analysis was performed using Matlab version 2014b (Mathworks, Natick, MA, USA).

#### NIRS

The O_2_Hb signal was filtered with a moving average filter of 10 s to remove high‐frequency noise. Next, we selected the recovery part of the O_2_Hb signal from end‐exercise until the maximum value was reached, and baseline‐corrected this by subtracting the end‐exercise value. This signal was fitted with a mono‐exponential model (eqn 1):

(1) $$\left[O_{2}Hb\left(t\right)\right]\;=\;\left[O_{2}Hb_{0}]+[\Delta O_{2}Hb\right]\left(1-e^{-k_{O2Hb}\cdot t}\right)$$

where *k*_O2Hb_ is the recovery rate constant, O_2_Hb_0_ is O_2_Hb at the end of exercise and ΔO_2_Hb is the recovery value of O_2_Hb minus O_2_Hb_0_.

#### IVIM

The TA was delineated on eight slices of the b = 0 s mm^−2^ image using MIPAV (http://mipav.cit.nih.gov) for all 16 diffusion‐weighted acquisitions to determine the average signal intensity for the TA per slice, b‐value and diffusion‐weighted acquisition. The ninth, most proximal, slice was excluded, because in most cases the TA was too small to be accurately delineated in the ninth slice. For each slice and diffusion‐weighted acquisition, the diffusion signal decay was fitted with a bi‐exponential model (eqn 2), in two steps (Le Bihan *et al*. 1988).

(2) $$S_{b}=S_{0}^{\prime }\;\left(\left(1-F_{p}\right)e^{-bD}+F_{p}e^{-b\cdot D^{*}}\right)$$

The diffusion coefficient (*D*) was computed by a linear least‐squared fit to the log‐transformed signal for b‐values ≥ 200 s mm^−2^ according to eqn 3.

(3) $$\log\left(S_{b}\right)=-Db+\log\left(S_{0}^{{}^{\prime \prime }}\right)$$

Thereafter, the perfusion coefficient (*D* ^*^), perfusion fraction (*F*_p_) and *S*_0_’ were fitted with a non‐linear least‐squared fit to eqn 2 with b‐values from 5 to 600 s mm^−2^ with fixed *D*. The fitted parameters *D*, *D* ^*^, *F*_p_, and the blood flow‐related parameter *F*_p_ × *D* ^*^ during rest and recovery were defined as the average over the first four and last 11 diffusion‐weighted acquisitions, respectively. The first acquisition after exercise was excluded, being often corrupted by motion artefacts.

#### ^31^P MRS

To assess the ^31^P signal coming from the TA and the extensor digitorum (ED) the contours of these muscles were delineated on the five slices of the T1‐weighted anatomical images and overlaid on the ^31^P images. Thereafter, the sum intensity of the ^31^P signals for both regions of interest was determined to calculate the relative contribution of the two dorsiflexors to the total ^31^P signal.

The ^31^P MR spectra were fitted, after phase correction and frequency alignment, using the AMARES algorithm in jMRUI (version 5.2, http://www.jmrui.eu) (Stefan *et al*. 2009) with Lorentzian line shapes, multiplets for ATP, and a singlet or doublet for Pi, as appropriate. Average PCr/Pi ratios were determined from spectra of the TA per coil element, assuming similar ^31^P T1 relaxation times distally and proximally. The PCr recovery was fitted with a mono‐exponential model (eqn 4).

(4) $$PCr\;\left(t\right)=PCr_{0}+\Delta PCr\left(1-e^{-k_{PCr}\cdot t}\right)$$

where *k*_PCr_ is the rate constant of PCr recovery, PCr_0_ is the PCr level at the end of exercise and ΔPCr is the recovery value of PCr minus PCr_0_. The parameter *t* is the time from the moment of the end of exercise. The relative amount of PCr depletion in exercise was calculated from the fitted values as ΔPCr/(ΔPCr + P_0_). The end‐exercise pH (pH_endex_) was determined from the chemical shift difference between Pi and PCr (Moon and Richards, 1973). In cases where two Pi peaks were fitted at end‐exercise, the average pH_endex_ was defined as the average pH of the two pH pools. Furthermore, we estimated the post‐exercise PCr resynthesis (V_PCr_) as *k*_PCr_×ΔPCr, because this directly reflects the initial post‐exercise O_2_ utilization (Kemp *et al*. 2015). For this calculation, ΔPCr is expressed in mM using an assumed ATP tissue concentration of 8.2 mM and applying T1 relaxation correction (T1_ATP_ = 5 s, T1_PCr_ = 6.6 s).

#### Carnosine

Signals for carnosine are observed in water‐suppressed ^1^H MRS spectra at 7 ppm and 8 ppm. To determine the relative tissue concentration, the 8 ppm peak was fitted with a Lorentzian line in jMRUI (version 5.2, http://www.jmrui.eu) and its amplitude was normalized to the amplitude of the water signal from the water‐unsuppressed ^1^H MR spectrum (Naressi *et al*. 2001; Stefan *et al*. 2009).

### Statistical analysis

We assessed the proximo‐distal variation in rest total 31P signal and PCr/Pi and post‐exercise *k*_O2Hb_, *D*, *F*_p_ × *D* ^*^, *k*_PCr_, and *V*_PCr_ along the TA in two ways with IBM SPSS Statistics (version 25, Chicago, IL, USA). First, the most distal position was compared with the most proximal position using a two‐sided paired samples *t* test for the continuous exercise and a Wilcoxon signed rank test for the intermittent exercise. Second, the dependence of PCr/Pi, *k*_O2Hb_, *D*, *F*_p_ × *D* ^*^, *k*_PCr_ and *V*_PCr_ on the location along the TA was tested with linear and non‐linear mixed models to account for intrasubject correlations (locations). For the linear mixed model, we included location as a fixed covariate (i.e. *y* = β_0_ + β_1_location), and for the non‐linear mixed model we included location and its quadratic term as fixed covariates (i.e. *y* = β_0_ + β_1_location + β_2_location^2^). In both mixed models, the intercept was modelled as a random effect, and the variance components covariance structure was used. For the evaluation of *k*_PCr_, the pH_endex_ was included as an additional fixed linear covariate with coefficient β_3_. All models were fitted using a maximum likelihood estimation, and the most appropriate model, linear or non‐linear, was chosen based on the Bayesian Information Criterion (BIC). The ratio of carnosine to water was compared between the distal and proximal voxel with a two‐sided paired samples *t* test. Moreover, a Pearson's correlation was used to estimate the association between *k*_PCr_ and *V*_PCr_ with *k*_O2Hb_ and *F*_p_ × *D* ^*^. As the data for the different measurement locations within a single volunteer show a high dependency, the requirement of the Pearson's correlation that all data points must be independent is not met. Therefore, we determined the average values of *k*_PCr_, *V*_PCr_, *k*_O2Hb_, and *F*_p_ × *D* ^*^ from all volunteers per coil element, optode position or image slice and correlated those average values instead of the individual data points. Data are presented as means ± SD unless stated otherwise.

## Results

### Subjects

The 15 healthy volunteers that performed a continuous isometric exercise were 26 ± 3 years old with BMI 22.8 ± 1.5 kg m^−2^ (Table 1). Their self‐reported activity ranged from 4 h to 69 h per week at various intensities (Table 1). The five additional volunteers doing intermittent isometric exercise were 31 ± 4 years old with BMI 21.3 ± 1.6 kg m^−2^. For the continuous exercise, some datasets had to be excluded. Two subjects were excluded from the IVIM analyses because of acquisition problems. Furthermore, five subjects were excluded from the ^31^P MRS analysis: in three subjects ^31^P MRS data were not acquired due to scanner problems, one subject showed no detectable PCr drop in two probe elements (possibly due to movement of the coil element towards the tibia) and in one subject the PCr recovery data could not be properly fitted with the exponential model because PCr did not recover for E1 and E2 (possible also due to coil movement).

**Table 1:** Subject characteristics – demographics and self‐reported physical activity for the 15 volunteers doing continuous isometric exercise

**Demographics**	
Age (years)	26 ± 3
Weight (kg)	76.1 ± 8.8
Height (m)	1.82 ± 0.08
BMI (kg m^−2^)	22.8 ± 1.5
**Self‐reported physical activity**	
Sitting (h:min per day)	7:18 ± 2:22 (3:51 to 11:09)
Walking (h:min per week)	4:58 ± 4:15 (0:30 to 16:40)
Cycling (h:min per week)	2:46 ± 2:36 (0:00 to 7:00)
Heavy intensity activities (h:min per week)	2:58 ± 2:35 (0:00 to 10:00)
Medium intensity activities (h:min per week)	5:48 ± 9:21 (0:00 to 36:00)

Data are presented as means ± SD for demographics and means ± SD (min to max) for self‐reported physical activity.

### MVC and force during exercise

In the continuous isometric exercise group, MVC was 200 ± 42 N for NIRS assessment and 203 ± 24 N for the MR assessment. During the isometric exercise for the NIRS, IVIM and ^31^P MRS measurements, the measured force was 28.6% ± 1.1%, 28.9% ± 0.7%, and 28.6% ± 0.8% of MVC and the average time to exhaustion was 296 ± 133 s, 250 ± 103 s, and 145 ± 46 s, respectively. The time to exhaustion during intermittent isometric exercise was 187 ± 4 s.

### NIRS

**Figure 2:**
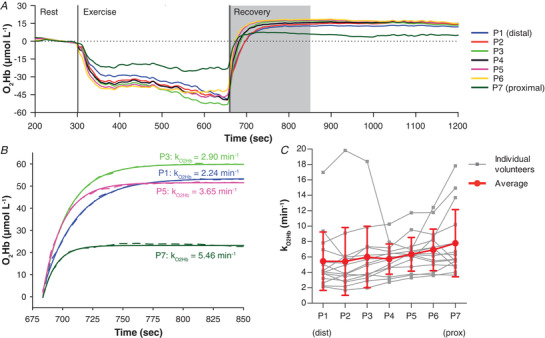
Time and spatial variations in the change in tissue oxyhaemoglobin (O_2_Hb) concentration assessed with near infrared spectroscopy (NIRS) in exercise experiments *A*, typical example of one volunteer for the change in tissue O_2_Hb concentration during rest, exercise and recovery. Data are shown from the last 100 s of rest to 1200 s after the start of the experiment. An expansion of the time window during recovery, indicated by the grey block, is in *B*. *B*, recovery of O_2_Hb, baseline‐corrected to end‐exercise values, during the first 170 s after exercise for optode position P1 (distal), P3, P5, and P7 (proximal) depicted as dashed lines. The corresponding mono‐exponential fit is depicted as solid lines. *C*, recovery rate constant of O_2_Hb (*k*_O2Hb_) for each volunteer per optode position and averaged over all volunteers (P1 distal, P7 proximal). The linear gradient in *k*_O2Hb_ was 0.15 min^−1^cm^−1^ (standard error: 0.04 min^−1^cm^−1^) Data are presented as means ± SD.

The O_2_Hb signal of the NIRS measurement, which reflects the imbalance between O_2_ supply and O_2_ utilization, was stable during rest and decreased as expected during continuous isometric exercise. After the exercise, the O_2_Hb signal recovered, often overshooting to a higher value than baseline (Fig. 2*A*). In the typical example presented in Fig. 2*B*, recoveries of the O_2_Hb signal are shown for the different optodes with recovery rate constants, *k*_O2Hb_, increasing from distal to proximal. On average over all subjects, *k*_O2Hb_, was 5.4 ± 3.8 min^−1^ at the distal optode and 7.8 ± 4.4 min^−1^ at the proximal optode (*P* = 0.011; Table 2, Fig. 2*C*).

**Table 2:** Outcome measures of NIRS, IVIM, ^31^P MRS and ^1^H MRS and cross‐sectional area along the length of the tibialis anterior muscle

	n	Distal	Middle	Proximal	*P* value distal *vs*. proximal
***Group continuous exercise – subjects 1 to 15***					
*NIRS*					
*k*_O2Hb_ (min^−1^)	15	5.4 ± 3.8	5.7 ± 2.0	7.8 ± 4.4	0.011
*IVIM*					
CSA (cm^2^)	13	3.7 ± 0.7	5.2 ± 0.9	2.7 ± 1.0	0.025
*D* (mm^2^s^−1^)	13	1.64 ± 0.04	1.62 ± 0.03	1.66 ± 0.05	0.063
*F*_p_ × *D* ^*^ (×10^−3^ mm^2^s^−1^)	13	0.77 ± 0.33	0.84 ± 0.14	1.12 ± 0.46	0.034
*^31^P MRS*					
CSA (cm^2^)	10	2.6 ± 0.8	5.9 ± 0.9	2.5 ± 0.9	0.712
PCr/Pi in rest	10	7.0 ± 0.7	8.3 ± 1.5	8.9 ± 0.9	*P* < 0.001
PCr depletion (%)	10	41 ± 7	54 ± 5	45 ± 10	0.269
pH_endex_	9	6.87 ± 0.10	6.74 ± 0.09	6.75 ± 0.12	0.033
pH1 (range; *n*)		6.88 – 7.07; 7	6.83 – 7.07; 4	6.76 – 7.03; 4	
pH2 (range; *n*)		6.71 – 6.88; 7	6.67 – 6.85; 4	6.61 – 6.89; 4	
*k*_PCr_ (min^−1^)	10	0.44 ± 0.26	0.50 ± 0.37	1.50 ± 0.57	<0.001
*V*_PCr_ (mM min^−1^)	10	5.2 ± 3.1	9.4 ± 8.3	23.3 ± 8.9	<0.001
***Group intermittent exercise – subjects 16–20***					
*^31^P MRS*:					
PCr depletion (%)	5	44 ± 3	46 ± 3	38 ± 3	0.068
pH_endex_	5	6.64 ± 0.21	6.65 ± 0.24	6.81 ± 0.20	0.138
pH1 (range; *n*)		6.99; 1	6.96 – 7.03; 2	6.79 – 7.02; 3	
pH2 (range; *n*)		6.79; 1	6.77 – 6.86; 2	6.63 – 6.80; 3	
*k*_PCr_ (min^−1^)	5	0.70 ± 0.65	0.68 ± 0.48	1.66 ± 1.00	0.043
*V*_PCr_ (mM min^−1^)	5	8.7 ± 8.1	9.2 ± 6.2	22.2 ± 14.2	0.043
***Group ^1^H MRS in rest – subjects 21–26***					
Carnosine (×10^−6^)^*^	6	1.74 ± 0.42		1.71 ± 0.48	0.884

Abbreviation: CSA, cross‐sectional area. Data are presented as means ± standard deviations. ^*^reflects the carnosine signal relative to water.

### IVIM

**Figure 3:**
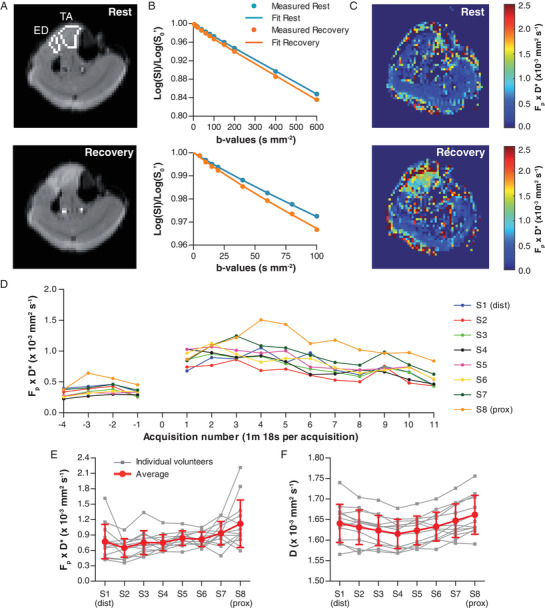
Results of IVIM acquisition of the lower leg for a typical example and average results in all volunteers *A*, diffusion‐weighted images at rest (top) and recovery (bottom, acquisition 6) of the middle slice (S5) for b = 5 s mm^−2^ with the tibialis anterior (TA) and extensor digitorum (ED) delineated. The TA and ED show an increased signal intensity after exercise. *B*, example fit of the IVIM model on data from the TA during rest (blue) and recovery (orange) for b_0_ to b_600_ (top) and b_0_ to b_100_ (bottom). *C*, blood flow‐related parameter *F*_p_ × *D* ^*^ map during rest (top) and recovery (bottom, acquisition 6) indicating an increased blood flow in TA and ED after exercise. *D*, *F*_p_ × *D* ^*^ over time for the eight analysed slices. Acquisition 0 is the first acquisition after exercise and excluded from the analysis because it is prone to movement artefacts. *E* and *F*, average *F*_p_ × *D* ^*^ and diffusion coefficient (*D*) over all volunteers depicted as the average over the whole recovery period after exercise (15 min 36 s) for the eight slices (S1 distal, S8 proximal). Data are presented as means ± SD.

The diffusion‐weighted MR images after continuous isometric exercise revealed an increased signal intensity compared with rest in the TA and ED, indicating that both muscles were activated during the exercise (Fig. 3*A*). For the low b‐values (0–100 s mm^−2^), the signal decayed faster post‐exercise than pre‐exercise, as depicted for the TA in Fig. 3*B*. This is reflected in an increased blood flow (*F*_p_ × *D* ^*^) in the TA and ED post‐exercise (Fig. 3*C*). Combining all volunteers, pre‐exercise values in the whole TA were 2.6% ± 0.5% for *F*_p_, 13.9 ± 2.2 × 10^−3^ mm^2^s^−1^ for *D* ^*^, 0.36 ± 0.10 × 10^−3^ mm^2^s^−1^ for *F*_p_ × *D* ^*^, and 1.57 ± 0.02 × 10^−3^ mm^2^s^−1^ for *D*. For the whole TA the values for *F*_p_, *D* ^*^ and *F*_p_ × *D* ^*^ increased significantly from rest to the recorded recovery period with 63% ± 37%, 58% ± 37% and 164% ± 108%, respectively (all *P* < 0.001). Also, the diffusion coefficient *D* of the TA significantly increased after exercise, with 4.0% ± 1.4% (*P* < 0.001), respectively. The increase in IVIM measures was most prominent in the first half of the number of acquisitions after exercise and recovered slowly to baseline during the second half (Fig. 3*D*).

The average *F*_p_ × *D* ^*^ over the whole recovery period after exercise (15 min 36 s) differed between the most distal and most proximal slice, from 0.77 ± 0.33 × 10^−3^ mm^2^s^−1^ distally to 1.12 ± 0.46 × 10^−3^ mm^2^s^−1^ proximally (*P* = 0.034) (Table 2, Fig. 3*E*). The *D* was lowest at the muscle belly with 1.62 × 10^−3^ ± 0.03 mm^2^s^−1^ and there was no difference between the distal and proximal slice (*P* = 0.063; Table 2, Fig. 3*F*). The cross‐sectional area of the TA for the eight slices was 3.7 ± 0.7 cm^2^, 4.5 ± 0.8 cm^2^, 5.0 ± 0.6 cm^2^, 5.3 ± 0.8 cm^2^, 5.2 ± 0.9 cm^2^, 4.8 ± 0.6 cm^2^, 4.1 ± 0.8 cm^2^, 2.7 ± 1.0 cm^2^ from S1 (distal) to S8 (proximal), respectively.

### ^31^P MRS

#### Rest

**Figure 4:**
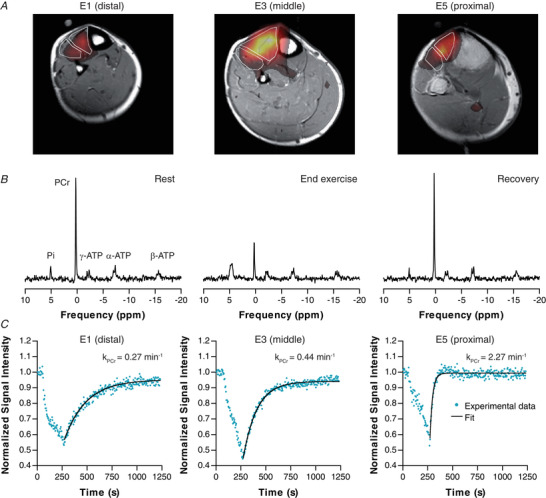
Example of ^31^P imaging, ^31^P spectra and PCr signal intensity time‐course *A*, overlay of ^31^P maps on T1 weighted ^1^H images, indicating that the majority of the ^31^P signal comes from the tibialis anterior. ^31^P intensity variations between slices may occur because of the ^31^P slice profile. *B*, ^31^P spectra showing inorganic phosphate (Pi), phosphocreatine (PCr) and the three resonances of ATP, and their change from rest to end‐exercise to end‐recovery. *C*, PCr signal time‐course for coil elements E1 (distal), E3 and E5 (proximal), showing a faster PCr recovery proximally. The PCr recovery rate *k*_PCr_ assessed from spectra of each coil element is indicated.

To establish the origin of the ^31^P MR signals received by the array coil we analysed ^31^P MR images overlaid on anatomical ^1^H MR images. The ^31^P signal (being mainly PCr) came for 58% ± 3% (mean ± SEM), 75% ± 3%, 79% ± 3%, 78% ± 2%, and 66% ± 3% from the TA for elements E1 to E5, respectively, confirming that the dominant proportion of the ^31^P signal originated from the TA (Fig. 4 *A*). The cross‐sectional area of the TA at the position of E1 (distal) to E5 (proximal) was 2.6 ± 0.8 cm^2^, 4.7 ± 0.8 cm^2^, 5.9 ± 0.9 cm^2^, 5.2 ± 0.9 cm^2^ and 2.5 ± 0.9 cm^2^, respectively. The summed ^31^P signal intensity from PCr + Pi + ATP did not differ (*P* = 0.550) between the distal (438 ± 81 × 10^3^ a.u) and proximal element (462 ± 80 × 10^3^ a.u) of the array coil. However, the phosphorylation potential, as reflected in the PCr/Pi ratio, was significantly lower in the distal element than the proximal element (7.0 ± 0.7 *vs*. 8.9 ± 0.9, *P* < 0.001, Table 2).

#### Continuous isometric exercise

For all five elements, the ^31^P spectra showed the expected drop in PCr signal and increase in Pi signal during exercise, with post‐exercise signal recovery of both, while the three resonances of ATP remained stable (Figs. 4*B*/4 *C* for E1/E3/E5). In the example illustrated, PCr recovered faster in the proximal element (E5) than in the distal element (E1). Combining all subjects and coil elements, the average PCr depletion was 48% ± 4%, pH_endex_ was 6.78 ± 0.09, and *k*_PCr_ was 0.73 ± 0.21 min^−1^.

**Figure 5:**
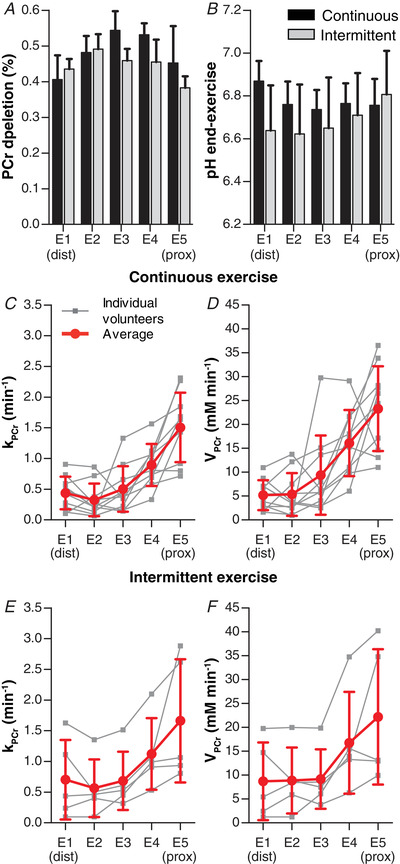
^31^P MRS results of the continuous isometric exercise and the intermittent isometric exercise for the coil elements E1 (distal) to E5 (proximal) *A*, PCr depletion for both exercise regimes. *B*, pH at end‐exercise (pH_endex_) for both exercise regimes. *C*, phosphocreatine recovery rate (*k*_PCr_) during continuous isometric exercise. *D*, estimated PCr resynthesis rate (*V*_PCr_ = *k*_PCr_ × ΔPCr) during continuous isometric exercise. *E*, *k*_PCr_ during intermittent isometric exercise. *F*, *V*_PCr_ during intermittent isometric exercise. Data are presented as means ± SD.

The assessment of intramuscular difference along the TA revealed that PCr depletion at the end of exercise was largest for the middle element E3 (54% ± 5%), but not different between the distal and proximal elements (*P* = 0.269) (Table 2, Fig. 5*A*). The corresponding pH at the end of exercise (pH_endex_) was higher distally than proximally (6.87 ± 0.10 *vs*. 6.75 ± 0.12, *P* = 0.033) (Table 2, Fig. 5*B*). To determine this pH_endex_, Pi was fitted as two peaks in 47% of all ^31^P spectra (22% of all distal *vs*. 56% of all proximal spectra) and pH was determined as their average. After exercise, the pH continued to drop for 30 s in all five elements after which it slowly restored. These temporal pH changes did not differ between the five elements. After exercise, PCr recovered slower (i.e. the rate constant was smaller) for the distal element compared with the proximal element (0.44 ± 0.26 *vs*. 1.50 ± 0.57 min^−1^; *P* < 0.001) (Table 2, Fig. 5*C*). The PCr resynthesis, *V*_PCr_, was lower distally than proximally (5.2 ± 3.1 *vs*. 23.3 ± 8.9 mM min^−1^; *P* < 0.001) (Table 2, Fig. 5*D*). To ensure that the fitted *k*_PCr_ was not corrupted by any movement of the coil, we performed consistency checks. First, we determined the sum of PCr and Pi from spectra obtained during the first 30 s post‐exercise relative to rest. The normalized PCr + Pi values were 0.90 ± 0.08, 0.86 ± 0.06, 0.90 ± 0.05, 0.92 ± 0.06 and 0.92 ± 0.09 for E1 to E5, respectively, indicating no movement effect. Furthermore, *k*_PCr_ was fitted with the first, the first four and the first eight data points removed. In all cases, *k*_PCr_ still displayed a proximo‐distal gradient similar to that shown in Fig. 5*C*, also indicating that no coil displacement occurred in the early phase of the recovery.

#### Intermittent isometric exercise

Averaged over the whole TA, intermittent isometric exercise caused a PCr depletion of 44% ± 3%, a pH_endex_ of 6.69 ± 0.20, and a *k*_PCr_ of 0.95 ± 0.59 min^−1^. The ^31^P MR spectra of the five coil elements showed that PCr depletion was, at 49% ± 4%, largest in element 2 (E2), but not different for the distal compared with proximal element (44% ± 3% *vs*. 38% ± 3%, *P* = 0.068) (Table 2, Fig. 5*A*). No significant difference in pH_endex_ was observed between the distal and proximal elements (6.64 ± 0.21 *vs* 6.81 ± 0.20, *P* = 0.138) (Table 2, Fig. 5*B*). This pH_endex_ was determined as the average of two Pi peaks in 60% of all ^31^P spectra (80% of all distal *vs*. 40% of all proximal spectra); in the other 40% of the spectra, pH_endex_ was determined from a single Pi peak. In line with the recovery after continuous exercise, both *k*_PCr_ and *V*_PCr_ were lower distally than proximally for the intermittent exercise (0.70 ± 0.65 *vs*. 1.66 ± 1.00 min^−1^; *P* = 0.043 and 8.7 ± 8.1 *vs*. 22.2 ± 14.2 mM min^−1^; *P* = 0.043, respectively) (Table 2, Figs. 5*E*/5*F*).

### Carnosine

**Figure 6:**
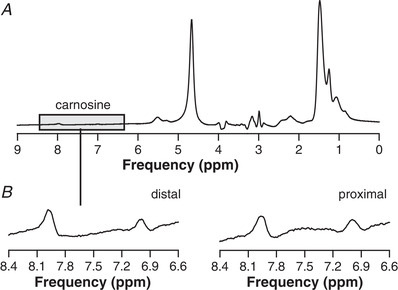
Example ^1^H MR spectra from tibialis anterior *A*, carnosine is represented by two peaks, at 7 and 8 ppm. Expansions of this spectral region from spectra of voxels positioned distally (B, *left*) and proximally (*B, right*).

In ^1^H MR spectra of all six volunteers, carnosine signals were visible in the distal and proximal voxel at 7 and 8 ppm (Fig. 6). The signal integral of the 8 ppm carnosine peak relative to the water signal at rest did not differ between the distal and the proximal voxel (1.74 × 10^−6^ ± 0.42 × 10^−6^ *vs*. 1.71 × 10^−6^ ± 0.48 × 10^−6^, *P* = 0.884, Table 2).

### Statistical outcomes of mixed model and correlation analysis

The outcomes of the statistical analysis with mixed models for the NIRS, IVIM and ^31^P MRS measurements during and following continuous isometric exercise are presented in Table 3. For the NIRS, the relation between *k*_O2Hb_ and the seven locations along the TA was best fitted with a linear mixed model, whereby optode location had a significant effect on *k*_O2Hb_ (*P* < 0.001).

**Table 3:** Estimates of the (non‐)linear mixed models and the corresponding ***P*** values

	BIC		Estimates of the (non‐)linear mixed model				*P* values		
	Linear mixed model	Non‐linear mixed model	β_0_ *(Intercept)*	β_1_ *(Location)*	β_2_ *(Location^2^)*	β_3_ *(pH_endex_)*	β_1_ *(Location)*	β_2_ *(Location^2^)*	β_3_ *(pH_endex_)*
**NIRS**									
*k*_O2Hb_	492	494	4.75 (0.85)	0.37 (0.09)			< 0.001		
**IVIM**									
*D*	‐460	‐503	1.66 (0.01)	‐0.020 (0.003)	0.003 (0.0003)		< 0.001	< 0.001	
*F*_p_ × *D* ^*^	8	4	0.80 (0.09)	‐0.07 (0.04)	0.013 (0.004)		0.087	0.003	
**^31^P MRS**									
PCr/Pi	137	138	6.8 (0.35)	0.44 (0.06)			<0.001		
*k*_PCr_	292	291	‐4.3 (3.9)	‐0.37 (0.19)	0.11 (0.031)	0.72 (0.56)	0.060	0.001	0.207
*V*_PCr_	348	347	6.2 (4.2)	‐2.5 (3.2)	1.20 (0.52)		0.444	0.028	
pH_endex_	‐71	‐74	6.97 (0.06)	‐0.13 (0.04)	0.018 (0.006)		0.002	0.008	
P_Cr_ depletion	‐99	‐120	0.25 (0.04)	0.18 (0.03)	‐0.027 (0.005)		<0.001	< 0.001	

According to the Bayesian Information Criterion (BIC), the *k*_O2Hb_ and PCr/Pi were best fitted with a linear mixed model and *D*, *F*_p_ × *D* ^*^, *k*_PCr_, pH_endex_, PCr depletion, and *V*_PCr_ were best fitted with a non‐linear mixed model. In the case of *k*_PCr_, pH_endex_ was also added as a fixed covariate. *P* < 0.05 indicates that the predictor (location, location^2^, or pH_endex_) has a significant effect on the fitted outcome measure (using type III F‐test). Data are presented as means (standard error).

The IVIM parameters *F*_p_ × *D* ^*^ and *D* were best fitted with a non‐linear model, with *P* values for location^2^ being *P* = 0.003 and *P* < 0.001, respectively. For location we obtained *P* = 0.087 and *P* < 0.001, respectively.

For the ^31^P MRS results of the TA at rest, the PCr/Pi ratio was linearly related to the coil element number (location: *P* < 0.001). The post‐exercise variables, *k*_PCr_ and *V*_PCr_ showed a significant non‐linear relationship with coil element number for the continuous isometric exercise (location: *P* = 0.060 and *P* = 0.444, location^2^: *P* = 0.001 and *P* = 0.028, respectively), which in the case of *k*_PCr_ could not be explained by the covariate pH_endex_ (*P* = 0.207). Furthermore, pH_endex_ and PCr depletion showed a significant non‐linear association with coil element number (location: *P* = 0.002 and *P* < 0.001, and location^2^: *P* = 0.008 and *P* <0.001, respectively).

For the intermittent isometric exercise, the mixed model did not converge, presumably due to the lack of power with only five subjects. Nevertheless, it did show a trend that *k*_PCr_, PCr depletion and *V*_PCr_ correlated with location, consistent with the results of the continuous isometric exercise.

**Figure 7:**
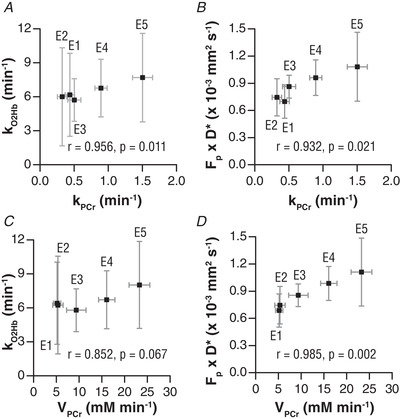
Phosphocreatine (PCr) recovery rate constant (*k*_PCr_) and PCr resynthesis rate (*V*_PCr_) displayed against the oxyhaemoglobin recovery rate constant (*k*_O2Hb_) and IVIM measured blood flow (*F*_p_ × *D* ^*^) A, k_PCr_ vs. k_O2Hb_. B, k_PCr_ vs. F_p_ × D^*^. C, V_PCr_ vs. k_O2Hb_. D, V_PCr_ vs. F_p_ × D^*^. Data are displayed per coil element as the average over all volunteers in means ± SD.

The correlation analysis revealed that *k*_PCr_ correlated strongly with *k*_O2Hb_ (*r* = 0.956, *P* = 0.011) and *F*_p_ × *D* ^*^ (*r* = 0.932, *P* = 0.021) (Figs. 7*A*/7*B*). The PCr resynthesis rate *V*_PCr_ also strongly correlated with *F*_p_ × *D* ^*^ (*r* = 0.985, *P* = 0.002) and showed a trend in the correlation with *k*_O2Hb_ (*r* = 0.852, *P* = 0.067) (Fig. 7 *C*/*D*).

## Discussion

In this study we investigated spatially specific interactions between O_2_ supply and consumption, and the generation of biochemical free energy (ATP, PCr) along the length of the working human TA. To examine this physiology *in vivo* we combined spatially resolved data measured by NIRS for O_2_Hb, MRI (IVIM) for perfusion and ^31^P MRS for energy metabolism before, during and after isometric exercise of this muscle.

We observed that following continuous isometric ankle dorsiflexion at 30% maximum force until exhaustion, the recovery rate constants of O_2_Hb (*k*_O2Hb_) and of PCr (*k*_PCr_), as well as perfusion (*F*_p_ x *D* ^*^), and the absolute rate of PCr recovery (*k*_PCr_ × ΔPCr) increased from distal to proximal along the length of the TA. As PCr resynthesis is proportional to the oxygen‐dependent suprabasal ATP synthesis, and as additionally O_2_Hb recovers faster proximally than distally, this indicates that O_2_ supply also exhibits a proximo‐distal gradient with the highest O_2_ supply proximally. Furthermore, we found that following intermittent isometric exercise *k*_PCr_ also increased from distal to proximal along the TA, indicating that prolonged ischaemia during exercise cannot explain the observed gradient in k_PCr_ and O_2_ supply following exercise. In contrast to these proximo‐distal gradients occurring upon exercise the relative carnosine content did not differ between the distal and proximal part of the TA, indicating no major variation in fibre type.

The recovery rate constant *k*_PCr_ is a well‐established marker of muscle mitochondrial function, reflecting maximal potential capacity for oxidative ATP synthesis (*Q*_max_). The *Q*_max_ derived from our ^31^P data showed a similar proximo‐distal gradient (data not shown). However, *Q*_max_ is a function based on severable variables and assumptions; hence we chose to focus on *k*_PCr_ instead, because it is simpler and more robust than *Q*_max_. When O_2_ supply is not limiting, *k*_PCr_ correlates with biopsy measures of mitochondrial content and function (Kemp *et al*. 2015). Studies typically compare *k*_PCr_ before and after an intervention, and/or between a disease and control group. In those studies, faster PCr recovery kinetics, indicative of greater ‘mitochondrial function’, represent better muscle performance. It is interesting to compare the spatially dependent *k*_PCr_ values in our study with those determined without an attempt to define the measurement location along the TA. In eight such studies reviewed in Kemp *et al*. (2015), the mean value is 1.35 min^−1^, which is closer to the proximal than the distal *k*_PCr_ values in the present study (see Table 2).

Intramuscular differences in *k*_PCr_ have also been observed in the gastrocnemius muscle (Niess *et al*. 2020). In contrast to our study, PCr recovery after exercise was faster distally than proximally and the difference was much smaller (1.25 min^−1^ *vs*. 1.00 min^−1^) than in the TA (0.44 min^−1^ *vs*. 1.50 min^−1^). These two studies are difficult to compare, because the 8 cm distal–proximal length studied in the gastrocnemius medialis corresponds to a coverage of only two of our adjacent ^31^P coil elements. Our results show that differences between two elements highly depend on where they are located along the muscle (see Fig. 5).

With the present study, applying continuous isometric ankle dorsiflexion at 30% MVC until exhaustion, we confirm our earlier observation of a substantial negative proximo‐distal gradient in *k*_PCr_ along the TA after ankle dorsiflexion, but performed at 60% MVC for 40 s (Boss *et al*. 2018). Moreover, this gradient in the TA was also observed after incremental exercise at several intensities until exhaustion and after intermittent isometric exercise.

The type of exercise might influence the spatial *k*_PCr_ gradient in two ways, via spatial variation in the degree of ischaemia caused by the isometric exercise and via end‐exercise pH. Ischaemia is not likely to play a major role, since the proximo‐distal gradient in *k*_PCr_ is essentially the same with intermittent isometric exercise, in which the contraction pattern has less scope for interfering with blood flow. Cytosolic acidification, measured by end‐exercise pH, has a complex effect on PCr recovery, tending to decrease *k*_PCr_ (Walter *et al*. 1997; van den Broek *et al*. 2007; Kemp *et al*. 2015). In the present study, the TA exhibited significant cytosolic acidification near end‐exercise, with the most severe acidification in the proximal part of the TA, where *k*_PCr_ was highest, clearly ruling out pH as a potential factor. During the intermittent exercise, by contrast, the lowest pH was found in the distal part of the muscle. However, the proximo‐distal pH difference of 0.2 is not large enough to explain a twofold proximo‐distal difference in *k*_PCr_ (Bendahan *et al*. 1990; van den Broek *et al*. 2007). In the 40 s submaximal exercise no pH drop was observed (Boss *et al*. 2018). In none of the cases does end‐exercise pH emerge as a significant factor in the mixed model analysis. Therefore, we can conclude that proximo‐distal variation in pH does not explain the proximo‐distal gradient in *k*_PCr_.

Is it possible that the spatial variation in *k*_PCr_ depends on a proximo‐distal gradient in O_2_ supply? The muscle has relatively little O_2_ storage capacity so the rates of net O_2_ supply and O_2_ usage are closely coupled. Net O_2_ consumption is the product of the arterial venous oxygen difference (AVD) and blood flow; thus for any rate of O_2_ use the O_2_ supply can be matched by appropriate changes in AVD or blood flow. Changes in AVD could in theory be assessed via amplitude changes in the NIRS deoxyhaemoglobin signal. However, these amplitude changes are highly dependent on the ratio between the active tissue volume and assessed tissue volume. This ratio is unknown and likely to vary along the TA, and therefore the change in AVD cannot be reliably assessed. We therefore have no direct evidence on AVD, but in the absence of large changes in AVD we would therefore expect absolute blood flow to track O_2_ supply and thus O_2_ consumption, spatially.

There has been much interest in proximo‐distal gradients in muscle blood flow and O_2_ supply and O_2_ use. Generally speaking, these studies are in line with the proximo‐distal gradients we found in *k*_O2Hb_ and muscle perfusion (*F*_p_ x *D* ^*^), both being lower in the distal part of the TA, suggesting lower O_2_ supply in distal than proximal muscle (Miura *et al*. 2001; Mizuno *et al*. 2003; Crenshaw *et al*. 2010; Niess *et al*. 2020). This is reflected in larger NIRS changes (deoxygenation) in the distal part of the vastus lateralis and gastrocnemius (Miura *et al*. 2001; Crenshaw *et al*. 2010). Furthermore, an MRI study using arterial spin label (ASL) showed a lower muscle perfusion more distally in the gastrocnemius (Niess *et al*. 2020). Direct comparison of these studies with our work are problematic as we investigated spatial variations along nearly the whole TA, covering 20 cm, while the coverage of the NIRS and ASL studies was 10 cm and 8 cm, respectively. Furthermore, for NIRS, these studies reported absolute recovery rates, while we assessed recovery rate constants. In a study using positron emission tomography ($H_{2}^{15}O$ PET), both blood flow and O_2_ uptake increased from distal to proximal in the quadriceps muscles during rest, but this gradient disappeared following exhaustive exercise (Mizuno *et al*. 2003). There appear to be no previous studies of this phenomenon in the human TA, but, agreeing with our study, the TA in the rat has a higher capillary density proximally than distally (Torrella *et al*. 2000).

There is no doubt that mitochondrial function as measured by ^31^P MRS and muscle blood flow are related. For example, there is a correlation between whole‐muscle *k*_PCr_ and post‐exercise perfusion determined by ASL (Duteil *et al*. 2004; Carlier *et al*. 2006). Furthermore, while whole‐muscle *k*_PCr_ in untrained subjects does not increase with an increase in the fraction of inspired oxygen, suggesting that O_2_ supply is not normally a limiting factor for PCr recovery, it does decrease when the fraction of inspired oxygen is decreased (Haseler *et al*. 2004, 2007).

If the primary cause of slower PCr recovery distally is a lower perfusion, limiting oxidative ATP synthesis, then we expect an increased average rate and extent of both deoxygenation and PCr fall during exercise (assuming no significant spatial gradients in ATP demand), followed post‐exercise by decreased rate constants of both PCr resynthesis and reoxygenation, in distal compared with proximal muscle (Kemp *et al*., 1995, 2001). According to the equation *V*_PCr_ = *k*_PCr_ × ΔPCr, given exponential recovery kinetics, a lower PCr resynthesis rate after the same or a greater fall in PCr amounts to a decreased PCr recovery rate constant (ratio of the two quantities (*k*_PCr_ = *V*_PCr_/ΔPCr). We do in fact observe a decreased *V*_PCr_ and *k*_PCr_ distally, but no difference in ΔPCr. By an analogous argument, a lower reoxygenation rate from the same or a lower state of oxygenation amounts to a decreased reoxygenation recovery rate constant (*k*_O2Hb_). We do see a decreased *k*_O2Hb_ in distal muscle. Across the spatial gradient, the rate constants *k*_PCr_ and *k*_O2Hb_ would change in the same direction, as we indeed observed. However, if the primary cause was vascular, the relative ‘abnormality’ (i.e. in distal relative to proximal) in reoxygenation would exceed that in PCr recovery: a pattern seen in e.g. moderate peripheral vascular disease (Kemp *et al*. 2001). But this is not what we see in the TA, where the differences in PCr recovery kinetics are proportionally twice as great as the reoxygenation kinetics. This makes it highly unlikely that lower perfusion in the distal part of the muscle explains the spatial variation in *k*_PCr_.

Consider now the alternate situation that the spatial variations in *k*_PCr_, *k*_O2Hb_ and IVIM perfusion measures are all determined by a lower intrinsic mitochondrial function distally. In general, in the face of impaired mitochondrial function, oxidative ATP synthesis rate can be maintained to some extent by classical closed‐loop feedback (increased fall in PCr, rise in [ADP], etc.) (Kemp *et al*. 2015). For a lower mitochondrial function distally, up to a certain energy demand, *V*_PCr_ can thus be maintained at the expense of a bigger ΔPCr, implying a lower *k*_PCr_ (*k*_PCr_ = *V*_PCr_/ΔPCr); beyond that energy demand *V*_PCr_ cannot be maintained and then *k*_PCr_ is even lower. What are the implications for NIRS kinetics? A lower distal *V*_PCr_ implies that O_2_ use is lower distally. If O_2_ supply is matched to O_2_ use, deoxygenation will be less distally and one might expect *k*_O2Hb_, therefore, to be higher distally. In fact, we observe that *k*_O2Hb_ is slightly lower distally. However, it can be questioned whether O_2_ supply and O_2_ use are exactly matched, since the NIRS recovery kinetics are dominated by O_2_ delivery rather than use of O_2_ (as post‐exercise O_2_ supply drives oxygenation state back to baseline). This makes it difficult to predict how *k*_O2Hb_ will behave in the situation of spatial variation of mitochondrial function. A higher intrinsic mitochondrial function proximally could be due to a higher mitochondrial content proximally (Park *et al*. 2014), but there are no existing data on the distribution of mitochondrial content along the human TA.

Note that lower intrinsic mitochondrial capacity might also explain the lower resting phosphorylation potential (PCr/Pi) observed distally. This increased distal feedback signal is necessary to maintain resting ATP synthesis rate despite a spatial variation in mitochondrial function operating across the whole dynamic range of muscle power (Kemp *et al*. 2015). Instead it is hard to explain lower resting phosphorylation potential in terms of reduced vascular O_2_ supply distally, which is not expected to be limiting at rest.

The similar PCr and pH decline proximally and distally during exercise, in particular during submaximal exercise (Boss *et al*. 2018), suggests that energy expenditure at both ends of the TA is not much different. So the question is what functional differences between proximal and distal parts of the TA are served by higher proximal rates of PCr recovery, perfusion and oxygenation after exercise? Proximally, the TA arises from the lateral condyle and upper lateral surface of the tibia and converges into a belly‐shaped fleshy muscle with a relatively large cross‐sectional area, while distally this area becomes smaller and at about one third of the tibia the TA is linked to a tendon. So this anatomical variance suggests that a more proximally higher contractile property is associated with faster post‐exercise energy recovery. Is this reflected in a proximo‐distal variation in fibre‐type distribution, organization or properties? It has been reported that the human TA consists of about 70% oxidative fibres, but to our knowledge there are no studies on fibre‐type distribution along the length of the TA in humans (Johnson *et al*. 1973). Our observation that tissue levels of carnosine, as a surrogate fibre‐type marker (Harris *et al*. 1998), are similar along the TA, suggests that a proximo‐distal variation in fibre type is not important, though this needs to be confirmed by more direct measurements. Studies in rats and rabbits found that the area occupied by oxidative fibres in TA increases from distal to proximal, indicating increased mitochondrial capacity (Wang and Kernell, 2001). The human TA has a bipennate fibre architecture in which the axial orientation of the aponeurosis varies along the length of the muscle and the pennation angle increases in the distal–proximal direction from 7° to 15° (Hiblar *et al*. 2003; Lansdown *et al*. 2007), although an earlier study reported no such variation in pennation angle (Maganaris and Baltzopoulos, 1999). Sarcomeres in fibres of the rat TA have different lengths between the distal and proximal parts of this muscle, of which the size depends on knee angle (Tijs *et al*. 2015). It has also been demonstrated that the TA may contain hybrid fibres with different myosin heavy chain expression (Medler, 2019). These different contractile structures may have different post‐exercise requirements for energy recovery. Finally, it may be argued that the lower distal *k*_PCr_ is due to more connective and fibrous tissue. However, these tissues do not contribute to the PCr signal involved in the *k*_PCr_ assessment and we observed that the (PCr + Pi + ATP) signal intensity is similar between the proximal and distal parts of the TA covered by the ^31^P array coil, indicating similar muscle content in these parts.

Whatever the underlying physiology, our work also has several methodological implications. The magnitude of the differences in *k*_O2Hb_, *F*_p_ × *D* ^*^ and *k*_PCr_ that we observed along the TA is comparable to differences in these variables observed in muscle diseases *vs*. healthy controls or the effect of training measured at a single muscle location. For instance, variations in *k*_O2Hb_ are in the order of the difference between healthy subjects and patients with chronic heart failure and peripheral vascular disease (McCully *et al*. 1997; Hanada *et al*. 2000), in *F*_p_ × *D* ^*^ the variations are as between the soleus and the gastrocnemius muscle (Mastropietro *et al*. 2018), and for *k*_PCr_, the differences are as between untrained and endurance‐trained TA (Larsen *et al*. 2009). This highlights the critical selection of a suitable measurement location and assuring reproducible repositioning in follow‐up studies for O_2_ supply and energy metabolic measurements, as a single location within the muscle might not represent the muscle as a whole. Furthermore, the significant variation in *k*_O2Hb_ and *F*_p_ x *D* ^*^ along the length of TA is in line with our previous muscle functional MRI (mf‐MRI) findings revealing a proximo‐distal gradient in the slope of the mf‐MRI signal increase after exercise (Boss *et al*. 2018). However, the mf‐MRI signal recovers 2–3 times slower than the NIRS O_2_Hb signal, suggesting that mf‐MRI reflects not only changes in oxygenation, but most likely also changes in T2 relaxation time due to other factors, like intra‐ and extracellular water shifts (Meyer and Prior, 2000; Damon and Gore, 2005; Schmid *et al*. 2014).

The present study was limited to young male subjects, in whom a thin subcutaneous fat layer facilitated a good SNR of the ^31^P MR and NIRS spectra in the TA. Future studies on whether this observed proximo‐distal gradient changes with age or training, and differs between women and men, could give additional information on the underlying mechanisms and muscle function. It is also of interest to assess whether the intramuscular variation in *k*_PCr_ occurs in other muscles such as the gastrocnemius and vastus lateralis, for which intramuscular variation in oxygenation and blood flow was already demonstrated. Mapping such intramuscular differences in multiple muscles could help to understand pathophysiological mechanisms in muscle disorders. For example, in muscular dystrophy patients, it is unknown yet why the disease spreads non‐uniformly along the proximo‐distal axis (Janssen *et al*. 2014; Hooijmans *et al*. 2017).

In conclusion, we provide evidence that in the human TA the post‐exercise O_2_ supply is higher proximal than distal, which is associated with a higher proximal PCr recovery rate constant *k*_PCr_ and resynthesis rate *V*_PCr_. Our experimental findings suggest that a higher intrinsic mitochondrial capacity could be the major factor underlying this proximal higher O_2_ supply and PCr recovery, apparently to serve a quick recovery of energy in the main contractile element of this muscle.

## Additional information

### Competing interests

All authors have nothing to declare.

### Author contributions

The experiments were performed at the department of Medical Imaging/Radiology of the Radboud University Medical Center, Nijmegen, The Netherlands. L.H., A.B. and A.H. were involved in the conception or design of the work. M.J.U built the ^31^P‐coil and helped to develop the experimental set‐up. L.H. and F.B. were involved in the acquisition and analysis of the data and drafting the work. M.F. provided help with the sequence optimization and analysis of the IVIM data. M.M. was involved in optimizing the IVIM sequence and in the statistical analysis. J.C. contributed to the NIRS data acquisition and analysis. L.H., F.L., G.K., A.B. and A.H. contributed to the interpretation of the work. All authors were involved in revising the work critically for important intellectual content.

All authors approved the final document and agreed to be accountable for all aspects of the work in ensuring that questions related to the accuracy or integrity of any part of the work are appropriately investigated and resolved. All persons designated as authors qualify for authorship, and all those who qualify for authorship are listed.

### Funding

L.H. was supported by the European Union's Seventh Framework Programme (FP7/2007–2013) under grant agreement number 305697. A.B. was supported by funding from the Centre for Systems Biology and Bioenergetics, Radboud University Nijmegen, the Netherlands.

## Supporting information


**Statistical Summary Document**

## Data Availability

The data that support the findings of this study are available from the corresponding author upon reasonable request.
